# The characteristics and impact of small and medium forest enterprises on sustainable forest management in Ghana

**DOI:** 10.1038/s41598-023-28403-8

**Published:** 2023-01-21

**Authors:** Stephen Anane, Emmanuel Kombiok, Alexander Baffour Afrifa

**Affiliations:** 1 Forestry Commission, Asankragua, Ghana; 2 University of Energy and Natural Resources, Sunyani, Ghana

**Keywords:** Climate sciences, Ecology, Environmental social sciences

## Abstract

Small and Medium Forest Enterprises are considered promoters of local development and growth due to their contributions to over 50% of forest-based employment in some countries like Brazil, Uganda, and India. Despite the many potentials of these enterprises, their operations go unchecked, which poses a serious threat to the sustainability of tropical forests. This study highlights the characteristics of SMFEs and the impact of SMFEs on sustainable forest management in Ghana from a survey of 80 randomly sampled enterprises in seven communities in the Amenfi West Municipality in Ghana. Data was gathered using structured questionnaires and key informant interviews and analyzed with statistical tools in SPSS version 25. The findings show that 71.25% of the SMFEs are not registered with the appropriate authorities like the Registrar General’s department and the Municipal Assemblies. In addition, 55 (68.8%) of these enterprises have a direct dependency on the forest for raw materials with 21.8% of this number claiming to have obtained the needed permits/licenses to harvest the raw materials they need from the forests. For 91.25% of the enterprises, factors such as resource availability and profits drive their activities and 8.75% are driven by access to labor and job creation. The analysis showed that belonging to an association is a significant determinant of business registration at p = 0.001. Forest policies must seek to promote sustainable management of forest resources by enforcing registration and permit laws. SMFEs must be consistently monitored and supervised to ensure that their activities are guided by policy and their compliance rewarded through capacity building and government support.

## Introduction

The concept of Sustainable Forest Management (SFM) is birthed from the strategies to mitigate the adverse impacts of deforestation with a special focus on developing countries^1^. The International Tropical Timber Organization aims to achieve this by reviewing, assessing, and monitoring forestlands through research to identify deforestation-causing factors in developing countries. It has been established that deforestation in developing countries is caused by illegal logging and trade, and Small and Medium Forest Enterprises (SMFEs) have been identified as culprits^2^.

The classification of forest enterprises is dependent on the volumes of sales, some employment, and capital investments. Legally, they are organized as cooperatives, producer associations, or conventional firms and they may have collective or private access to forest resources. SMFEs are mostly characterized by closeness to a resource base and objectives that include employment, income generation, and community development. In instances of favorable environments and markets, they extend their activities to value-adding such as finished wood products, sawn wood, processed NTFPs, and ecotourism^3,4^.

The World Bank approximates that 90% of the poorest people are heavily reliant on forests for income and subsistence. Therefore, SMFEs and their related development provide a favorable platform for strengthening the people's livelihood while conserving the natural resource base through sustainable forest management and value addition of non-timber forest products (NTFPs)^5,6^. In the context of technical and financial resources and political connections needed for effective maneuvering through bureaucratic processes and landmines, SMFEs are often inadequate in triggering a change in the political-legal framework. Though the extent of application of regulations varies across countries, generally, laws regarding forest management facilitates preferential access to large-scale forest enterprises more than SMFEs.

SMFEs are therefore established as a source of livelihood for their owners. However, their activities, have both expected and unexpected consequences on the environment and social development. Considering this, the study set out to identify the characteristics of SMFEs and evaluate their contributions to the Amenfi West Municipality with a specific focus on job creation and development. Due to the lack of strict enforcement of the regulations meant to check illegal logging, competition among SMFEs is not usually fair, as the domestic markets are compromised with illegal products^7^. According to Agrawal et al.^7^, often with the complicity of local government agents, 50–90% of extracted volumes in developing countries like Cameroon and Ghana are traded illegally. This highlights the need to assess the impacts of SMFEs on the sustainable management of Ghana’s forest resource management.

## Materials and methods

### Study area

**Figure 1:**
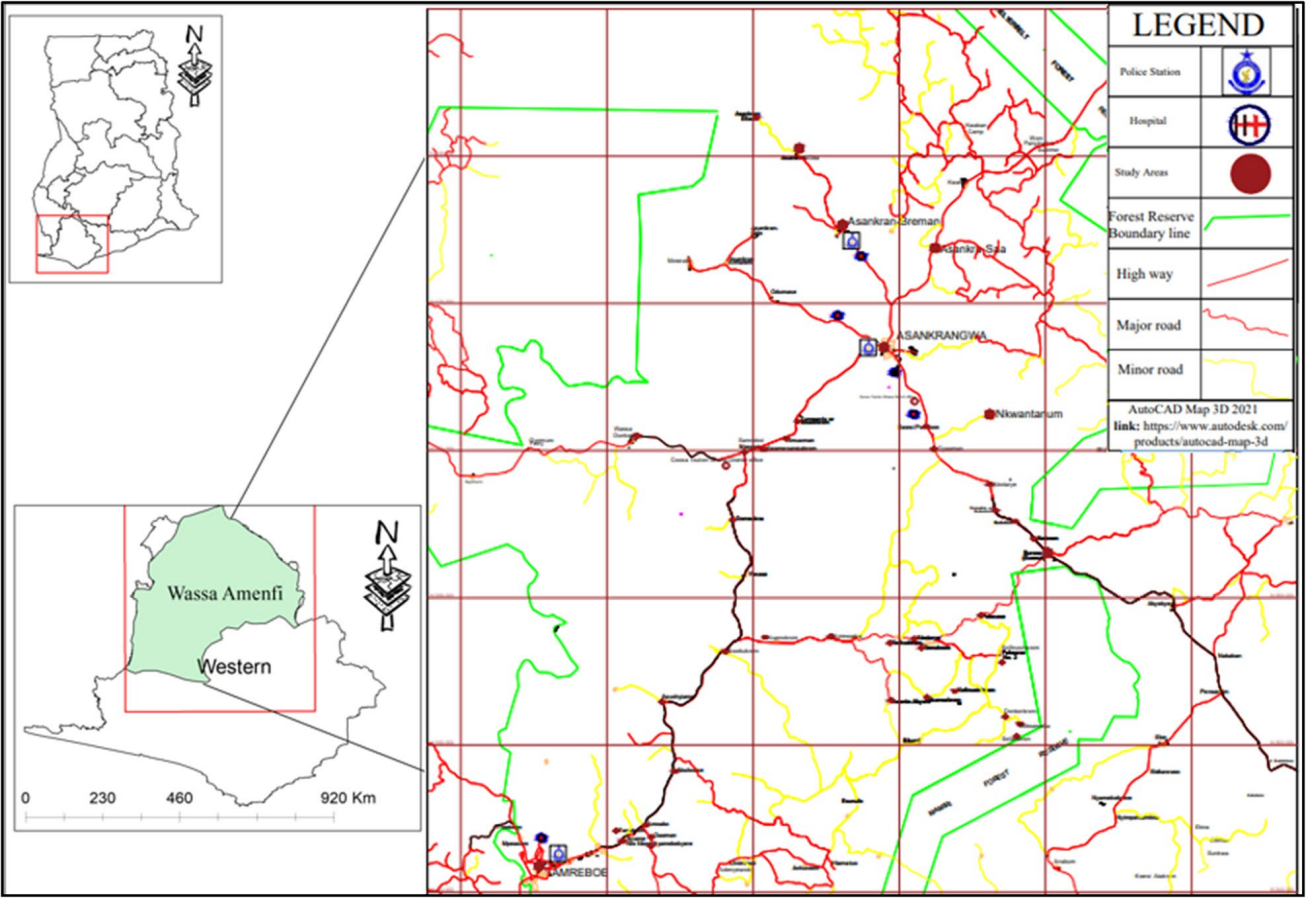
Map of the Amenfi West Municipal and Study areas.

The study was conducted in the Amenfi West Municipality, in the Western Region of Ghana (Fig. 1). The area is one of four agricultural zones in the region characterized by the vegetative cover that gives a vivid expression of the interaction between heavy rainfall and soil types. The Northern part holds the semi-deciduous forest whereas the south holds the tropical rainforest characterized by heavy rainfalls of about 1750 to 2000 mm according to the Ghana Statistical Service (GSS)^8^. An area of 64,242.81 hectares is covered by forest reserves^8^.

### Research design and sampling techniques

The case study approach was used to carry out the study. The approach as a scientific inquiry offers a systematic pathway for collecting in-depth information or investigating a person’s circumstance, a group, an institution, a community, or an incident/event^9^. This approach also permits the deployment of techniques, and methods such as interviews, participant observation, and field studies.

Probability and non-probability sampling techniques were used to select the respondents for this study. In this study, a simple random sampling method was employed in probability sampling to choose 80 respondents from seven communities. Non-probability sampling, on the other hand, utilized purposive sampling to select institutional representatives from key institutions to provide key information that were deemed essential for achieving the study’s goals. Two representatives each from Forestry Commission and National Board for Small Scale Industries were selected as key informants for interview. Table 1 provides a breakdown of the selected areas and sampled respondents.

**Table 1:** Sample Size Distribution of SMFEs by Location.

Selected areas	Sample frame	Sample size	
	Numbers SMFEs	Selected	Percentage (%)
Asankragwa	453	21	26.25
Asankran Saa	122	6	7.5
Asankran Breman	80	4	5
Samreboe	365	17	21.25
Nkwantanum	267	13	16.25
Sereso	112	5	6.25
Mumini	309	14	17.5
Total	1708	80	100

### Data collection and analysis

Data for this study were collected through interviewer-administered questionnaires and this was facilitated by interaction with respondents in a guided manner to enhance the collection of relevant data. Quantitative data was collected using structured household questionnaires whereas qualitative data were gathered through face-to-face interviews of institutional representatives with the aid of guides.

The questionnaire used in gathering the data consisted of four sections. Section A solicited information on the demographics of the respondents and section B gathered data on the characteristics of the SMFEs. Section C gathered data on the contributions of SMFEs to development and finally, section D looked at sustainability challenges in forest management relative to the activities of SMFEs. The questionnaire was pretested using 10% of the sample size and adjustments were made in the framing of questions to ensure easy understanding.

Following the pretest and finalization of the questionnaire, it was administered to the 80 randomly selected respondents. The collected data were coded and entered into SPSS (version 26) for analysis. Statistical tools like correlation, linear and logistic regressions were used to investigate the statistical relationships between variables to establish the needed empirical evidence for analysis.

All experimental protocols were approved by the Committee on Human Research Publication and Ethics (CHRPE) of the Kwame Nkrumah University of Science and Technology. In addition, all methods were carried out in accordance with relevant guidelines and regulations. Informed consent was obtained from all respondents.

## Results

### Demographic characteristics of SMFEs

**Figure 2:**
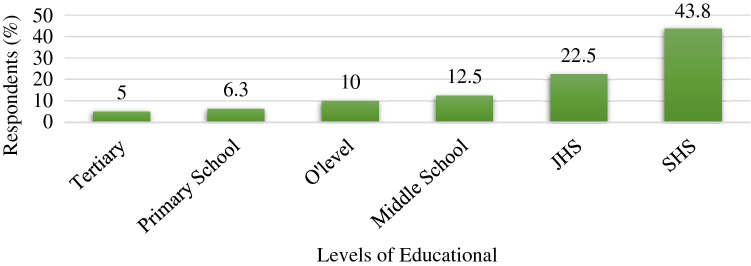
Educational History of Respondents.

The study showed that only 6.3% of the respondents were female and the remaining 93.7%, were male. In terms of education, the study revealed that all respondents had formal education, which ranged between primary and tertiary education levels. Only 5% had attained the tertiary level of education whereas 43.8% had Senior Secondary/High School level education (Fig. 2).

### The types of SMFEs in the study area

**Figure 3:**
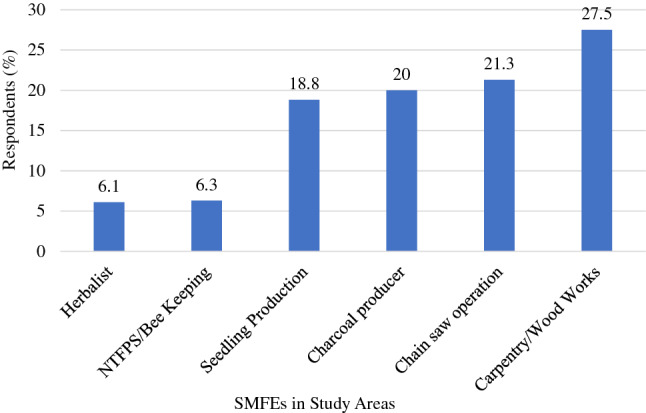
Various SMFEs in the Study Areas.

The study found a diverse number of SMFEs in terms of wood and non-wood-related activities. Six main types SMFEs were identified of which three were into wood-related activities and the other three into non-wood-related activities. Carpentry/Woods SMFEs were 27.5% whereas Herbalist/Herbal Practicing SMFEs were the least at 6.1%. Figure 3 provides the details of the distribution of respondents across the types of SMFEs identified in this study.

### SMFEs as a source of income

A major characteristic of a thriving economy is the availability and competitiveness of markets for goods and services. The study found many similarities between SMFEs in terms of composition and operation and therefore there is a need for them to adopt different strategies in advertising their products. The most adopted strategy by the respondents to improve their visibility included advertisement (45%), After Sales Promotion, Sales Promotions, and other strategies at 31.3, 22.5 and 1.3% respectively. The expenditures and sales of SMFEs contribute to the inflows and outflows of capital in the municipality. It was observed that the majority (72.5%) of the sales made by SMFEs were between $184 and $500 whereas 53.8% of the expenditures made were $17–$167.

The data showed that the overall total sales made were relatively higher than the overall total expenses, which suggests that these SMFEs are making profits. Table 2 reports a regression analysis of expenses against sales that showed a positive linear association (r = 0.680). The coefficient of determination (R^2^ = 0.462) indicates that 46.2% of the variance in monthly sales made is attributed to the monthly expenses of the SMFEs. Summarily, the expenditures as input capital for SMFEs reflect in their revenue positively, however, does not take into account other expenditures such as taxes.

**Table 2:** Linear regression of sales and expenditure.

Variable	Coefficient	Std. Error	t-statistic	Prob
C	− 0.380	0.244	− 1.559	0.123
Monthly expenditure	0.903	0.110	8.185	0.000***
R-squared	0.462			
Adjusted R-squared	0.455			
S. E. of regression	0.496			
F-statistic	66.986			
No. of observations	80			

Dependent Variable: Monthly Sales.

***Significance level at 1%.

### Employment creation by SMFEs

The level of employment in an economy is a key characteristic in determining the performance of that economy. The survey revealed that 81.25% of the SMFEs employed between 1 and 10 people, 15% employed 11–20 people, and 3.75% employed 21–30 people. The findings of this study as presented in Table 3 are in line with the definitions of SMEs in general.

**Table 3:** Breakdown of Employment in SMFEs.

Number of People	Frequency	Percent
1–10	65	81.25
11–20	12	15
21–30	3	3.75
Total	80	100

### Registration and tax obligations of SMFEs

As a requirement for running a business in Ghana, the business is required to register with the relevant authority. Furthermore, depending on the type of business, it may be necessary to register with a regulatory authority that is responsible for ensuring that the business’s products do not pose any risks to public health or the environment by adhering to specific production standards. This registration is required by law.

**Figure 4:**
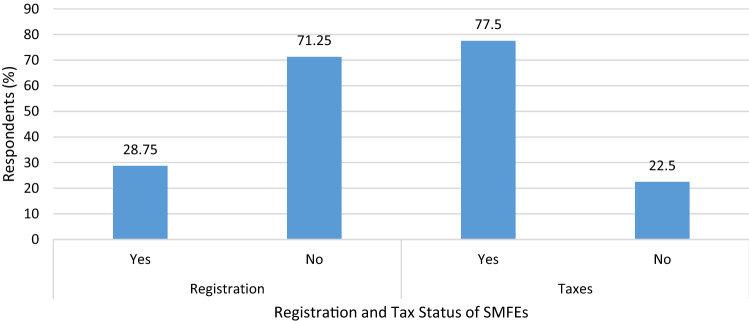
Registration and Tax Payments of SMFEs.

This study found that majority (71.25%) of the SMFEs have not registered with the relevant authorities as required of them. The study further revealed that of the 23 SMFEs representing 28.75% of the total SMFEs sampled that were registered, five, representing 21.74% were awarded certificates as Small Medium Enterprises whereas the remaining 78.26% obtained Small Scale Enterprise certificates. This study also found that 77.5% of the SMFEs pay taxes whereas 22.5% admitted to not paying taxes (Fig. 4).

Further investigation on the 77.5% that indicated that their businesses pay taxes revealed the various payments in terms of revenues made. The study showed that of the 62 businesses that paid taxes, 58.8% paid between $17 and $24. On the other hand, 15% paid between $34 and $83, and only 3.8% paid in the range of $85–$167 as taxes. It is key to note that, the amounts the SMFEs pay is solely income taxes. The calculation is solely based on the revenues generated by these businesses.

Table 4 reports the determinants of SMFE registration. The analysis showed that belonging to an association is a significant determinant of business registration at p = 0.001. Meanwhile, sex, age, and education level attained did not determine the registration of SMFEs. Associations are known to have rules and regulations or even constitutions that govern them and therefore registration of your business may be a requirement for any SMFE that wants to join. This also serves as a characteristic of their legitimacy since they would not want to be perceived as engaging in illegalities by associating with illegal or unregistered businesses. Correlation analysis showed a weak negatively significant relationship between payment of taxes and educational background at p = 0.030 whereas sex and age did not exhibit any significant relationship with payment of taxes (Table 5).

**Table 4:** Regression of Determinants of Business Registration.

	B	S.E	Wald	df	Sig	Exp(B)
Sex	17.332	17,428.85	0.000	1	0.999	33,650,826
Age	− 0.47	0.946	0.247	1	0.619	0.625
Education	0.513	0.479	1.151	1	0.283	1.671
Belong to an association	5.877	1.776	10.948	1	0.001***	356.693

***Significance level at 1%.

**Table 5:** Relationship between Sociodemographic Characteristics and Payment of Taxes.

Variables					
		Sex	Age	Educational background	Payment of taxes
Sex	Pearson correlation	1			
	Sig. (2-tailed)				
	N	80			
Age	Pearson correlation	0.028	1		
	Sig. (2-tailed)	0.808			
	N	80	80		
Educational background	Pearson correlation	0.074	− 0.089	1	
	Sig. (2-tailed)	0.512	0.434		
	N	80	80	80	
Payment of taxes	Pearson correlation	0.108	− 0.070	− 0.243*	1
	Sig. (2-tailed)	0.339	0.536	0.030	
	N	80	80	80	80

*Correlation is significant at the 0.05 level (2-tailed).

### Sources of raw materials for SMFEs

SMFEs depend on both wood and non-wood materials for their products and all of these come from the forests however, this happens directly or indirectly. Some SMFEs go directly to the field to acquire the raw materials using their labor. Some also depend on secondary sources to get their raw materials from places like sawmills and chainsaw operators after they have directly sourced the materials from the forests. From the results of the study, 68.8% of SMFEs directly access their raw materials from the forests. In addition, 7.4% (Table 6) depend on state institutions like Forestry Commission and Forest Research Institute of Ghana (FORIG) for their raw materials. By their nature, SMFEs are highly dependent on forest resources for operation as defined in their name as well.

**Table 6:** Sources of Raw Materials for SMFEs.

Source of raw materials	Frequency	Percent
Forest	55	68.8
Chainsaw operators	14	17.5
Sawmills	5	6.3
Forestry and FORIC	6	7.4
Total	80	100

Of the 55 (68.8%) SMFEs that directly source raw materials from the forest, only 12 representing 21.8% claim to have applied for permits/licenses to harvest raw materials from the forests (Table 7).

**Table 7:** SMFEs with Permits/Licenses for Harvesting Raw Materials.

Permit/License	Frequency	Percent
Permit for harvesting	12	21.8
Illegal harvesting	43	78.2
Total	55	100

### Factors driving the activities of SMFEs

A majority of the SMFEs (91.25%) cited factors such as resource availability and profits as major drivers of their activities. However, 8.75% of the SMFEs indicated dimensions such as labor and employment creation as the factors driving their existence. The results are presented in Table 8.

**Table 8:** Factors driving the activities of SMFEs.

Factors	Frequency	Percent
Resource availability	73	91.25
Labor and employment	7	8.75
Total	80	100

### Sustainability challenges in forest management relative to SMFEs activities

This study found that 71.25% of SMFEs were operating without registration, which makes it easier for their activities to go unchecked (Table 9). It also becomes impossible to regulate the frequency of forest entry by these SMFEs. To further ascertain the extent to which the activities of SMFEs affect SFM caused the investigation of four factors. These were the observation of standard precautions and protocols for harvesting materials, supervision of activities, knowledge of laws/regulations of the forest, and training on raw materials harvesting. The results of the study indicate that there is a lot to be done to minimize the negative impact of SMFEs activities on SFM. It was observed that the majority of the SMFEs do not observe the standard protocols put in place to ensure the non-destructive use of the forests.

**Table 9:** Factors Undermining Sustainable Forest Management concerning SMFEs.

Factors investigated	Response	Frequency	Percent
Observation of protocols/precautions	Yes	19	23.8
	No	61	76.3
Supervision of activities	Yes	12	15.8
	No	68	84.2
Knowledge of laws/regulations	Yes	16	20
	No	64	80
Training on raw materials harvesting	Yes	13	18.6
	No	67	81.4

## Discussion

### The contributions of SMFEs to the local economy and development

SMFEs are characterized by limited resources, hence their inability to employ more people however, the few being employed to aid in the operations of the businesses contribute to the reduction of the employment gap among the youth in the study areas. The employment opportunities provided by SMFEs supplement the central government’s efforts to offer employment to the people. Subsequently, the people in the area depend on it for their livelihood to improve their living standards. The study found a diverse number of SMFEs in terms of wood and non-wood-related activities (Fig. 3) that people engage in as primary or secondary jobs. Some evidence has proved that SMFE’s contribution to forest employment is above 50% in some countries like Brazil, Uganda, Guyana, and China, and almost 80 to 90% of all forest-based enterprises in most countries^7^. This may directly impact efforts to reduce poverty by improving the living standards of people who form and operate SMFEs as a livelihood.

Zada et al.^10^ also reported that households who own SMFEs had a wealth index increase from 5.4 to 7.4 whereas those without SMFEs had an index of 4.9. SMFEs do have the potential to improve household income levels which can lead to reinvesting and expansion. This study found that the monthly expenses of SMFEs contribute to 46.2% of their monthly sales. Therefore, if SMFEs can increase significantly, their ability to reinvest while observing the best practices in operating their businesses then they will be able to maximize their turnovers. This can result in expansion and more employment opportunities for others hence, reducing the burden on the government to provide employment.

There is a direct positive and significant relationship between SMFEs and local economic development^11^. SMFEs were reported to positively and significantly mediate the relationships between government support, entrepreneurship knowledge, and local economic development. SMFEs with informal or formal training can ensure government support is efficiently used in tapping into entrepreneurial knowledge to drive their impacts on local economies. This will also allow them to grow into sustainable businesses while also promoting the sustainability of forest resources which they depend on for raw materials.

### Operational characteristics and impacts of SMFEs on sustainability

Around the globe and per the laws of Ghana, businesses are required to fulfill certain obligations to enable them to run smoothly^12^. Failure to undertake these tasks may attract severe penalties, including criminal charges that may carry significant jail terms. An example is failure to pay taxes and adhere to certain regulations. This section looks at certain characteristics of SMFEs in this study that project their impact on sustainable forest management.

Firstly, the laws of Ghana make it mandatory for all business categories to pay tax and as such, SMFEs are not left out. However, the major challenge with taxes in Ghana and by extension, the world, is compliance. Mantey^13^ reported that 59.1% of small business owners did not understand the Ghanaian Tax System. The key lesson drawn from this observation is that, as SMFEs, one of their characteristics is that they generate a smaller income compared to larger companies or corporate bodies. This goes a long way in determining the amount they pay as taxes. In addition, by their nature, they can under-declare the revenue they make to influence the amount they will be taxed. This calls for the development and flawless implementation of mechanisms to monitor and audit these SMFEs to ensure that they comply with tax directives and regulations.

Mantey^13^ further reported that 57.4% of the surveyed business owners are not aware of most tax laws and guidelines on the taxation of incomes for organizations. Some blamed their inability to pay taxes on the business being slow and others were unwilling to give a response to why they were unable to pay their taxes. In this study, majority (77.5%) indicated that they pay taxes. It was also established in this study that payment of taxes has a significantly weak correlation with the educational background of the respondents. Though the majority of the SMFEs paid taxes, it may not be directly linked to all respondents having some form of formal education and vice versa. However, this may be factored in when considering the training and mentoring of SMFEs to contribute to local development by paying their taxes. More SMFEs may endeavor to pay their taxes regularly if they understand what these taxes can do to improve their work environment.

Governments in recent times have stepped up revenue mobilization efforts to capture more businesses into the tax bracket of the country. This has seen the revenue authorities recruit and train more revenue officers to reach businesses like SMFEs which are mostly not reachable due to their inability to register their businesses.

Secondly, the majority (71.25%) of SMFEs in this study was not registered. This adds to the general belief that most businesses operate without the required licenses or have failed to renew their expired licenses. Some studies also made similar observations and arrived at the lack of enforcement of laws, as a key reason why many businesses in developing countries remained unregistered contrary to the requirements of the law^14,15^. Further analysis showed SMFEs who belong to associations are likely to register their businesses because it is a requirement to join them. The benefits of belonging to an association include access to loan facilities and other credit programs and therefore some SMFEs do not want to risk missing out through failure to get their business registered^16^.

SMFEs need to get registered for them to be considered legitimate business entities however, this seems to be a challenge in most developing countries. Tomaselli et al.^17^ found this assertion relevant when investigating SMFEs access to microfinance. Registration of business is a key requirement to access loan facilities and so is belonging to a recognized association. Associations are known to serve as guarantors for members who want loan facilities from banks and other financial institutions to expand their businesses^16^. Unregistered, unregulated, and unmonitored SMFEs are those whose activities tend to compromise the sustainability of forest resources^18^. Therefore, registration of SMFEs does that only serve the interest of governments but also the interests of these SMFEs themselves.

The third has to do with the sourcing of raw materials. Ghana being a tropical country is blessed abundantly with forest resources but over the decades, the overexploitation of these forests has brought to the brink of extinction, various species of both plant and animal life^19^. The dependence of SMFEs on the forests cannot be underestimated as literature, citing Osei Tutu et al.^20^, posits that SMFEs contribute to 95% of the income of some rural households. This study shows that 68.8% of SMFEs get their raw materials directly from the forest. Both woody and non-woody materials are in abundance and can be extracted with minimal cost.

In sourcing raw materials from the forests in Ghana, SMFEs are required to obtain permits or licenses from the relevant authorities such as the forestry commission. This permit/license is what allows or gives this SMFEs access to otherwise inaccessible forest reserves to harvest raw materials^20^. Additionally, these documents can go as far as determining the type and quantity of materials to harvest. It can also determine the type of access granted as these accesses can vary or differ depending on the time or season of harvest^18^. The issuance of permits and licenses is meant to monitor and regulate resource harvesting with the primary goal of checking the overexploitation of these resources. However, this is not possible due to the high levels of non-compliance by SMFEs^21^. Evident in this study is the 78.2% of SMFEs who gather raw materials from the forest without permits/licenses.

Osei Tutu et al.^18^ concluded that the neglect of the SMFEs sub-sector is responsible for the loss of state revenue because of their unwillingness to register and pay appropriate taxes and permit fees for their illegal and unsustainable business operations. The report further posits that “despite the numerous support channels (national and international) available to them, the roles played by SMFEs in poverty reduction are significantly unimpactful hence the need to intensify capitalizing on all opportunities to address challenges they present.” The government institutions in charge of these forest resources depend on these permits and license fees to supplement their already insufficient government subventions for the operations. Therefore, losing revenues may undermine their sustainability programs.

### Driving factors of SMFEs

The ability of a business to thrive highly depends on its ability to overcome certain challenges within its operating environment^22^. That alone, however, is not enough as certain factors ignite the ambition of a business. These factors decisively influence the success or the failure of the business hence, they are identified as determinants. The study sought to identify some determinants that drive the activities of SMFEs. Responses from the SMFEs concluded that economic and social factors such as resource availability, profits/revenue, employment, and labor are the key determinants that drive the SMFEs.

Resource availability was the major driver of their activities cited by 91.3% of SMFEs. This is because, the numerous forests the nation is endowed with provide abundantly, the raw materials needed for them to use. Due to the favorable climatic conditions prevailing in the high forest zones, there is a constant supply of materials needed by SMFEs to produce their products for business^23^. In addition, availability means less competition for limited resources and therefore it boils down to the ability to process these raw materials into finished goods for market consumption hence, reducing the costs of production^24^.

SMFEs also pointed to profits/revenue, as the factor driving their activities to engage in, and sustain their business. The abundance and readily availability of raw materials are very important to the growth of their business and in turn, help them maximize their returns. This is because the inputs they make to acquire the raw materials are relatively low in comparison to the total revenues they generate. This observation is also reflected in the captured expenditures they make as inputs or investments into their businesses.

SMFEs that need technologically advanced mechanisms and equipment are those that are required or inclined to make heavy investments whereas those that need simple tools and equipment invest less. Whichever the case, the nature of SMFEs suggests that a business that requires raw materials with very minimal or no costs involved at all, yet yields very high profits, is how people can improve their living^25^. Badini et al.^26^ classified enabling environment of SMFEs into external and internal factors where financial capital, business management, and organizational capacities form internal factors. On the other hand, external factors include regulatory frameworks, forest law enforcement, and natural capital which refers to the stock of natural resources or environmental assets. The success of any SMFE is largely dependent on these factors.

Finally, 8.75% of the SMFEs view labor and employment, as the determinants driving their existence. For them, compared to other labor-intensive ventures, their business does not require huge labor to get work done. The few hands needed means most of the revenues do not go to paying workers. They can dictate and bargain to their advantage because there are many people without jobs hence a job turned down, because of less encouraging benefits is gladly accepted by another^25^. Ultimately, the study finds that labor is cheap in some areas of the SMFEs’ environs primarily, due to unemployment.

### Sustainability challenges in forest management relative to SMFEs activities

Since the United Nations Conference on Environment and Development (UNCED) in Rio de Janeiro, Brazil in 1992, key challenges of SFM have broadly covered the sustainability of forest resources through the reduction of deforestation and forest degradation, conservation and protection of biological diversity, genetic resources sustainability and improving forest goods and services valuation^27^. It is important to note that SMFEs have played an overlooked role in these challenges as it seems its contributions to poverty reduction have taken center stage in international discourses, with its negative impacts on the environment being relegated to the backseat when considering the causes of environmental degradation. Attempts to effectively manage the activities of SMFEs have witnessed the emergence of a lot of challenges that threaten the very sustainability the globe yearns for. Some reasons point to the source of the challenges that have plagued these efforts, some of which are highlighted below.

First is the lack of resources to recruit and train the needed personnel to constantly monitor the activities of these SMFEs during the harvesting of raw materials. This makes it easier for them to enter restricted forest areas without the necessary documentation and proceed to harvest more than they are required to at any given time. Secondly, it is difficult to track their activities because many SMFEs currently, do not register their businesses as required by law.

A typical example is the use of unapproved trails or routes and the use of inappropriate harvesting techniques such as burning. This leads to the destruction of various lifeforms that are critical to the regenerative capabilities of the forests^28^. The study also found that the supervision of the activities of SMFEs is very poor as only 12% of SMFEs had their activities supervised on certain occasions. This buttresses the assertion by Acheampong et al.^29^ who posited that the lack of supervision is a major issue that needs to be vigorously addressed if we need to achieve forest sustainability in developing countries.

There is a need to educate SMFEs on the laws and regulations governing the use of forest resources. It was revealed that only 16% of the respondents have some knowledge of the regulations governing the harvest and use of both woody and non-woody forest resources. This knowledge gap is being exploited by SMFEs as an excuse for not doing what is expected of them. However, a study found that 69% of respondents claimed to have good knowledge of the regulations governing their activities^14^. This can be attributed to self-learning or the action of the supervising authorities who for one reason or another other can perform their mandate of educating the SMFEs. There is a need to properly equip the supervising agencies to carry out this mandate.

The research, therefore, cites the non-registering of SMFEs as an underlying cause of the flouting of these regulations and laws. The research also suggests that some form of training can be done at the point of registering even before the certification is done. As observed in the area of training, there is not enough emphasis on the need to train SMFEs in sustainability issues in terms of harvesting raw materials. It was noted that the majority (67%) of SMFEs (Table 8) have no training on how to harvest, process, and adequately market their products to ensure maximum profits while sustaining the resources for future harvests. There is a need to institute training and capacity-building programs for SMFEs that will empower them to succeed and yet aim to ensure sustainable forest management.

### The role of sustainable forest management in climate change mitigation

Sustainable forest management (SFM) can play a significant role in climate change mitigation, as forests are an important sink for carbon dioxide and other greenhouse gases. By sequestering carbon in their biomass and soils, forests can help to remove carbon dioxide from the atmosphere, which can help to mitigate the impacts of climate change^30^.

There are a number of ways in which SFM can support climate change mitigation, including through the conservation and expansion of forests, the sustainable management of forests, and the use of forest-based products and practices that reduce greenhouse gas emissions. Policymakers and stakeholders at local, national, and international levels are increasingly recognizing the role of forests in climate change mitigation, and there is growing interest in developing strategies and policies that support the use of forests for this purpose.

However, there are challenges that impede the efficient leveraging of SFM for climate change mitigation and one of such challenges is the need to balance economic, social, and environmental considerations^31^. Forests provide a range of goods and services that are vital for human well-being and economic development, including timber, non-timber forest products, and ecosystem services such as carbon sequestration, water regulation, and habitat for wildlife^32^. However, these resources can be in high demand, and managing forests sustainably can be difficult, particularly in developing countries where there may be limited access to financial and technical resources^33^.

Another challenge is the impact of external factors such as climate change on the health and productivity of forests^34^. Rising temperatures and changing weather patterns can affect the growth and survival of forests, and may also increase the risk of forest fires and pests^35^. Policymakers must consider the role of forests in mitigating and adapting to climate change, as well as the potential impacts on forest-dependent communities^32^.

One way in which SMFEs can contribute to climate change mitigation is through the sustainable management of forests. By practicing sustainable forestry, SMFEs can help to maintain and enhance the carbon sequestration capacity of forests, which can help to remove carbon dioxide from the atmosphere and mitigate the impacts of climate change^31^. This can involve practices such as planting and reforestation, soil and water conservation, and the use of sustainable harvesting techniques^32^. However, this study revealed the majority of these SMFEs are unregistered and therefore not monitored. Meaning their activities cannot be regulated to ensure practices that promote climate change mitigation.

SMFEs can also contribute to climate change mitigation by using forest-based products and practices that reduce greenhouse gas emissions. For example, the use of wood products as a substitute for fossil fuel-based products can help to reduce emissions, as wood products sequester carbon over their lifetime and do not release it into the atmosphere when they are used^34^. In addition, the use of biomass energy in place of fossil fuels can help to reduce emissions, provided that the biomass is sourced sustainably and the emissions associated with its transportation and use are accounted for^35^.

Another way in which SMFEs can contribute to climate change mitigation is through the development of innovative solutions and technologies that support sustainable forestry practices and reduce greenhouse gas emissions. This could include the use of precision forestry techniques, which use advanced technology to improve the efficiency and sustainability of forestry operations^34^. It could also involve the development and commercialization of new forest-based products or practices that have a lower carbon footprint^32^.

Policies can have a significant impact on the way in which forests are managed for climate change mitigation^31^. For example, policies that promote sustainable forestry practices, such as the use of certification schemes or incentive programs, can help to ensure that forests are managed in a way that meets the needs of current and future generations^33^. On the other hand, policies that do not adequately consider the needs and interests of all stakeholders, or that do not provide sufficient support for sustainable forestry practices, may have negative impacts on the ability of forests to contribute to climate change mitigation^34^.

Overall, addressing the inter-challenges of SFM for climate change mitigation and the impact of policies is an important part of ensuring the sustainability and long-term viability of forests as a tool for mitigating climate change.

### Development of SMFEs within the forest-based economy of Ghana through policy

Despite the global consensus on the sustainability of forest resources and their utmost importance regarding the sustenance of present and future generations, the situation remains unclear at the field level^36^. The application of criteria and indicators of sustainability provides support for a small but crucial clarification on achieving sustainable forest management (SFM). A meaningful basis for assessing SFM at operational levels will require clarification together with management prescriptions and performance standards while providing linkage to voluntary timber certification.

Currently, many environment-based non-governmental organizations (ENGOs) like Global Footprint Network and Fauna & Flora International who are concerned about natural resource exploitation, are convinced by the international debate on criteria and indicators that timber harvesting and ecosystem services of the forests can be sustained^37^. Stakeholders of the forestry franchise agree that environmental conservation can be accommodated through a necessary and reasonable modification and adaptation of forest-harvesting practices. Therefore, multi-resource forest management as a new paradigm replaces the indigenous sustained-yield management approach that bases on growth-harvest equilibrium using policy as a vehicle^38^.

Food and Agriculture Organization (FAO) is assisting countries through policy advice, technical assistance, capacity building, workshop, and hands-on training, to overcome the challenges of sustainable forest management^39^. The assistance is provided through the assessment of forest resources and the elements of SFM, as well as the monitoring of progress toward it. FAO also identifies, tests, and modern scientific SMF approaches and techniques to address climate change mitigation and adaptation challenges such as increasing demand for wood and non-wood forest products and services, pest, and diseases.

The views held by the Forestry Commission and National Board for Small Scale Industries (NBSSI) during interviews are in line with the suggestions and actions by the World Bank and FOA that involve training and other support systems for managers of forest resources in tropical countries like Ghana that depends heavily on its natural forests. Despite the availability of some of the avenues needed to execute these strategies, the non-compliance by SMFEs makes it difficult for these targets to be met. The general thought is that, if all relevant authorities and stakeholders perform their roles effectively, the current challenges of maximizing the contributions of SMFEs to development and sustainable forest management can be realized.

The impact of forest policies is evident in countries like Gabon, a country rich in forest resources, which regards forests as a critical economic resource. World Bank-supported reforms have helped make concessions awarding procedures more competitive and transparent^40^. Forest taxation recovery has been bolstered, with tax collection rates increasing from 40 to 80% between 2005 and 2010. Sustainable forest management is presently practiced in around 85% of productive forest areas and as a result of these reforms, the forestry sector’s contribution to Gabon’s GDP increased from 2.5% in 2004 to 4.7% in 2009^40^.

Support for small and medium forest-based firms raised actual cash income among forest user groups by 53% in India’s Andhra Pradesh throughout the project duration. Seasonal outmigration decreased by 23%, and the quality of thick forest cover in these places improved^40,41^.

Ghana has made significant progress toward sustainable management of its forest resources via the adoption of different forest regulations like the Forest and Wildlife Policy of 1994, Timber Resources Management Act, of 2002, etc. The problem with most of the country’s forest resource policies is the lack of attention paid to the human component; the emphasis is on sustainable timber extraction, even if it is destructive to the livelihoods of forest-dependent populations. Forest policies have historically been determined by successive administrations' economic interests, which essentially focused on the exploitation of wood resources for income production. This has been a significant impediment to the creation and development of non-timber forest products which the majority of SMFEs depend on in Ghana. This has allowed the number SMFEs rapidly increase due to the lack of coverage by forest policies^42^. The policy interventions in Gabon and India have yielded results that can provide the foundation needed for Ghana to formulate its policies for the development of SMFEs in a way that does not threaten sustainable forest management.

## Conclusion

The contributions of SMFEs to local development are apparent to be noticed and diverse. Some of these contributions are in the areas of employment, balanced ecosystems, and inputs for the wood sector and foreign exchange. There is also available evidence that SMFEs can perform better in a forest context given the right conditions such as multiplicity of policy and institutional frameworks. The potential of SMFEs for local development notwithstanding, the persisting challenges compared to non-forest SMEs are complex. The security of tenure is a major challenge especially when one SMFE is unable to assert its right to the natural and forest resources with competitors. SMFEs provide mainly local jobs. However, SMFEs are noted for illegal logging and harvesting, largely due to poor legal frameworks. Although the activities of SMFEs although has some prospects for Ghanaians their grievous limitation is the blare nature of the existing regulatory framework governing the environment within which they operate. Thus, finding a lasting solution to this challenge among others stated in this research would significantly propel a good foundation upon which they can grow and develop their potential. Finally, this research concludes that sustainable forest management is beset with a plethora of challenges considering that most SMFEs are not registered, unlicensed to harvest raw materials, and non-compliant with tax laws and regulations.

## Data Availability

Available upon request from the corresponding author.
